# Genotypic variation in genome-wide transcription profiles induced by insect feeding: *Brassica oleracea *– *Pieris rapae *interactions

**DOI:** 10.1186/1471-2164-8-239

**Published:** 2007-07-17

**Authors:** Colette Broekgaarden, Erik H Poelman, Greet Steenhuis, Roeland E Voorrips, Marcel Dicke, Ben Vosman

**Affiliations:** 1 Plant Research International B.V., Wageningen University and Research Centre, Droevendaalsesteeg 1, 6708 PB Wageningen, The Netherlands; 2 Laboratory of Entomology, Wageningen University, P.O. Box 8031, 6700 EH Wageningen, The Netherlands

## Abstract

**Background:**

Transcriptional profiling after herbivore attack reveals, at the molecular level, how plants respond to this type of biotic stress. Comparing herbivore-induced transcriptional responses of plants with different phenotypes provides insight into plant defense mechanisms. Here, we compare the global gene expression patterns induced by *Pieris rapae *caterpillar attack in two white cabbage (*Brassica oleracea *var. *capitata*) cultivars. The two cultivars are shown to differ in their level of direct defense against caterpillar feeding. Because *Brassica *full genome microarrays are not yet available, 70-mer oligonucleotide microarrays based on the *Arabidopsis thaliana *genome were used for this non-model plant.

**Results:**

The transcriptional responses of the two cultivars differed in timing as characterized by changes in their expression pattern after 24, 48 and 72 hours of caterpillar feeding. In addition, they also differed qualitatively. Surprisingly, of all genes induced at any time point, only one third was induced in both cultivars. Analyses of transcriptional responses after jasmonate treatment revealed that the difference in timing did not hold for the response to this phytohormone. Additionally, comparisons between *Pieris rapae*- and jasmonate-induced transcriptional responses showed that *Pieris rapae *induced more jasmonate-independent than jasmonate-dependent genes.

**Conclusion:**

The present study clearly shows that global transcriptional responses in two cultivars of the same plant species in response to insect feeding can differ dramatically. Several of these differences involve genes that are known to have an impact on *Pieris rapae *performance and probably underlie different mechanisms of direct defense, present in the cultivars.

## Background

In nature, plants are constantly surrounded by herbivorous insects that negatively influence plant fitness. To effectively combat them, plants have evolved direct and indirect defense mechanisms [1-3]. Chemical compounds that play a role in direct defense are produced and stored in tissues of the plant that are consumed by herbivores [4,5]. These compounds can alter the physiology of herbivores by reducing their growth rate, adult size, and survival probability [5]. Glucosinolates, for example, are well characterized defense compounds of cruciferous plants that are hydrolyzed by specific thioglucosidases called myrosinases. This reaction results in the release of an array of toxic compounds such as isothiocyanates [6] that reduce herbivore survival, growth, and development rate [7]. In contrast to direct defense mechanisms, indirect defense mechanisms promote the effectiveness of the natural enemies of herbivores e.g. through volatile secondary metabolites [8,9]. Direct and indirect defense mechanisms can function additively against an herbivore. A slower herbivore growth can prolong the time that the herbivore is exposed to a predator or parasitoid [10]. Kessler and Baldwin (2004) showed that a combination of direct and indirect defense mechanisms of *Nicotiana attenuata *resulted in additional mortality of *Manduca sexta *larvae. Direct and indirect defense mechanisms can be constitutively present or induced upon herbivore attack [1,11].

Inducible defense mechanisms involve the activation of a set of genes in response to herbivore attack. DNA microarrays are excellent tools to elucidate the role of these genes in plant defense [12,13]. These tools have been extensively exploited to investigate inducible defenses in *A. thaliana*. *Pieris rapae *feeding in *Arabidopsis thaliana*, for example, induces more than 100 genes that are potentially involved in defense [14]. Additionally, similar expression patterns in response to feeding by *P. rapae *and *Spodoptera littoralis *caterpillars have been found [14]. Mechanical damage induces a different transcript profile than *P. rapae *feeding [15]. Attack by the phloem feeding aphid *Myzus persicae *results in the differential expression of many more genes than feeding by the caterpillar *P. rapae*: 2181 versus 186 genes [16].

Despite the availability of several accessions of *A. thaliana*, the studies on *A. thaliana*-insect interactions mentioned above have been performed for only one genotype (Columbia-0). No comparative information is available on the natural variation of global transcriptional responses of different genotypes within one species of the Brassicaceae family.

The most important signal-transduction pathway involved in inducible defense mechanisms of plants against chewing-biting insects is the jasmonate pathway [17]. Jasmonates are a family of lipid regulators that include jasmonic acid (JA), an oxylipin signaling molecule derived from linolenic acid [18]. JA accumulates in response to insect attack, resulting in the regulation of distinct sets of genes [14,16]. Studies in *A. thaliana *and tomato mutants deficient in JA synthesis or JA perception demonstrated that JA is essential for defense against some insects and mites [19-23]. Accumulation of JA can also be evoked by mechanical wounding alone [15].

Here, we compare the transcriptional responses of two *B. oleracea *cultivars upon feeding by larvae of *P. rapae*. Genes regulated in response to this chewing-biting insect were identified using an *A. thaliana *70-mer oligonucleotide microarray. These microarrays have been demonstrated to be effective for analyzing global gene expression in *B. oleracea *[24]. We aimed at characterizing genes that are potentially involved in inducible direct defense by comparing transcriptional responses of the white cabbage cultivars Rivera and Christmas Drumhead. In addition, the contribution of jasmonate-dependent and jasmonate-independent genes in the response of *B. oleracea *to *P. rapae *attack was investigated. Our results show the existence of clear genotypic differences in direct defense and in transcriptional responses between cultivars of *B. oleracea*.

## Results

### Larval performance on cultivars Rivera and Christmas Drumhead

**Figure 1:**
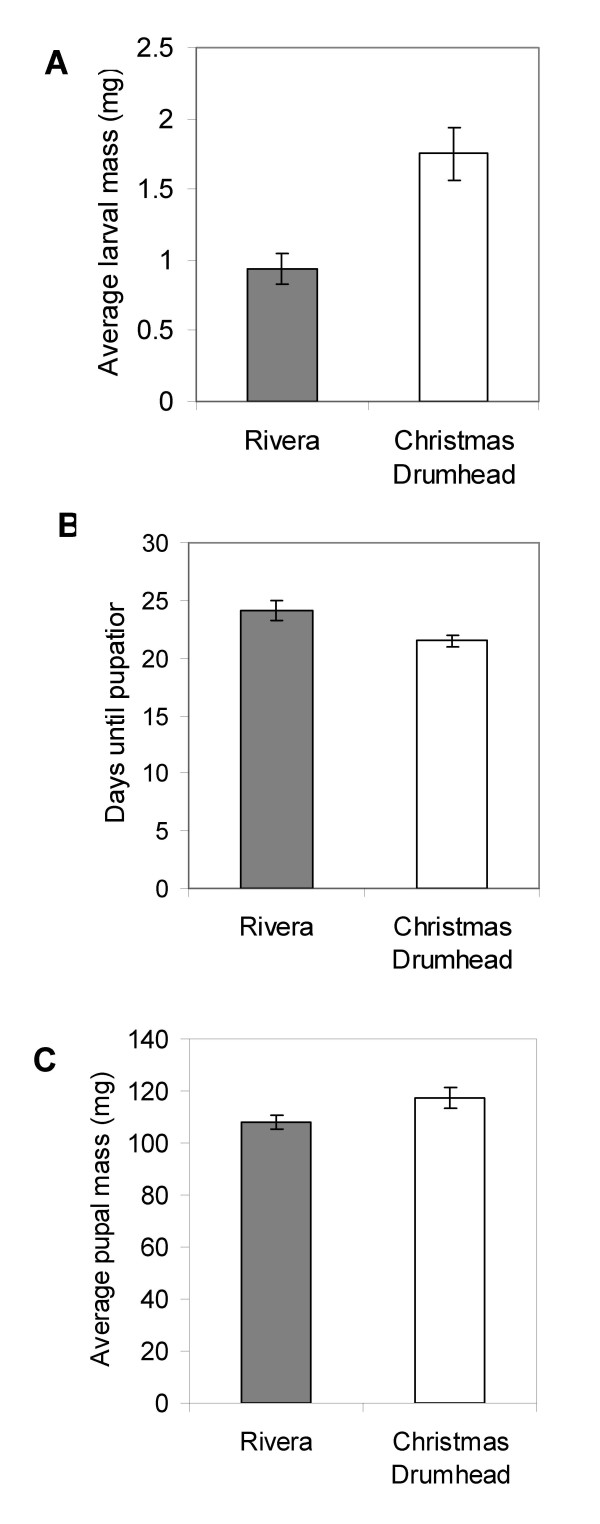
**Performance of *P. rapae *larvae on two *B. oleracea *cultivars**. A, Larval weight (mean + SE) after 6 days of feeding. B, Time to reach pupation (mean + SE). C, Pupal weight (mean + SE) just after pupation.

The white cabbage (*Brassica oleracea*) cultivars Rivera and Christmas Drumhead were characterized for larval performance of *P. rapae*. We found that *P. rapae *larvae feeding on Rivera had a significantly lower weight after six days than those feeding on Christmas Drumhead plants (Mann-Whitney U test, P = 0.001) (Figure 1A), indicating slower growth of *P. rapae *larvae on Rivera. Larvae feeding on Rivera pupated around 2.5 days later than those feeding on Christmas Drumhead plants (P = 0.005) (Figure 1B). Such retardation in developmental period has large consequences for population growth rates [25]. However, larvae feeding on either cultivar did not differ significantly in pupal weight (P = 0.376) (Figure 1C). The results showed that direct defense against *P. rapae *larvae was more pronounced in Rivera than in Christmas Drumhead plants.

### Statistical analyses of *P*. *rapae*-regulated genes in cultivars Rivera and Christmas Drumhead

Because Rivera and Christmas Drumhead displayed different levels of direct defense against *P. rapae *larvae, transcriptional responses to feeding by this insect species were monitored to identify genes that may contribute to inducible direct defense. For this purpose, microarray analyses were performed in which genes were considered to be differentially expressed when they showed an expression ratio ≥ 2-fold or ≤ 0.5-fold with a statistical significance of P < 0.05 (Student's *t *test).

For several genes the induction was highly significant (P < 0.01), although their expression change was between 1.5 and 2 fold. On the other hand, a number of genes showed at least a twofold change in all three replicates, but a P-value above 0.05 because of the large variation between replicates. These genes are potentially interesting candidates that would require careful investigation to determine whether their expression changes have biological relevance. However, these potentially interesting candidates were not considered as differentially expressed in this study.

### Transcriptional responses of cultivars Rivera and Christmas Drumhead to *P*. *rapae *feeding

**Figure 2:**
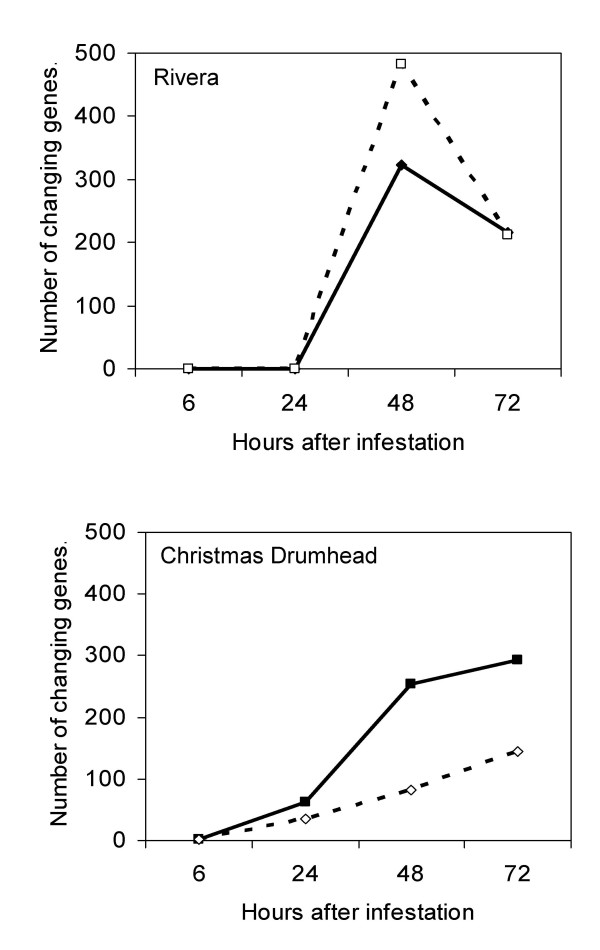
**Gene expression changes in cultivars Rivera and Christmas Drumhead after *P. rapae *feeding**. Number of expressed genes induced (closed symbols and solid line) and repressed (open symbols and dashed line) more than twofold and with P < 0.05 at the time points tested.

When comparing unchallenged plants with plants that had been attacked by *P. rapae *for 24 h, 99 genes had at least a two-fold change in expression level with a P value below 0.05 in Christmas Drumhead. Of these 99 genes, 63 were induced and 36 were repressed (Figure 2B). Remarkably, no genes met our selection criteria for induction or repression in Rivera after 24 h of *P. rapae *attack, although two genes showed an expression ratio ≥ 2-fold in two replicates and almost 2-fold (1.9) in the third replicate. These potentially induced genes included *Lipoxygenase2 *(At3g45140) and a gene encoding a trypsin/protease inhibitor (At1g72290). Both genes were significantly induced in Christmas Drumhead (Additional file 1). Based on these results, we hypothesized that Rivera has a slower transcriptional response than Christmas Drumhead upon attack by *P. rapae*. To test this hypothesis, we analyzed expression changes in both cultivars after 48 h of *P. rapae *infestation. Indeed, we identified many differentially expressed genes in Rivera at this time point, consisting of 322 induced and 483 repressed genes (Figure 2A). Many differentially expressed genes were also identified in Christmas Drumhead after 48 h of *P. rapae *feeding. In this cultivar, 254 induced and 83 repressed genes were identified (Figure 2B). After 72 h of *P. rapae *attack, 215 genes were induced and 213 repressed in Rivera (Figure 2A). In Christmas Drumhead, the number of differentially expressed genes after 72 h of caterpillar feeding increased to 292 induced and 144 repressed genes (Figure 2B). When the larvae had fed for only 6 h, we did not find any genes to be differentially expressed in Rivera according to our selection criteria. In Christmas Drumhead, we only found a gene encoding a trypsin/protease inhibitor (At1g72290) to be induced at this time point (Additional file 1). This suggests that after 6 h of larval feeding regulation of expression had not yet started or was not yet strong enough to be detected.

**Figure 3:**
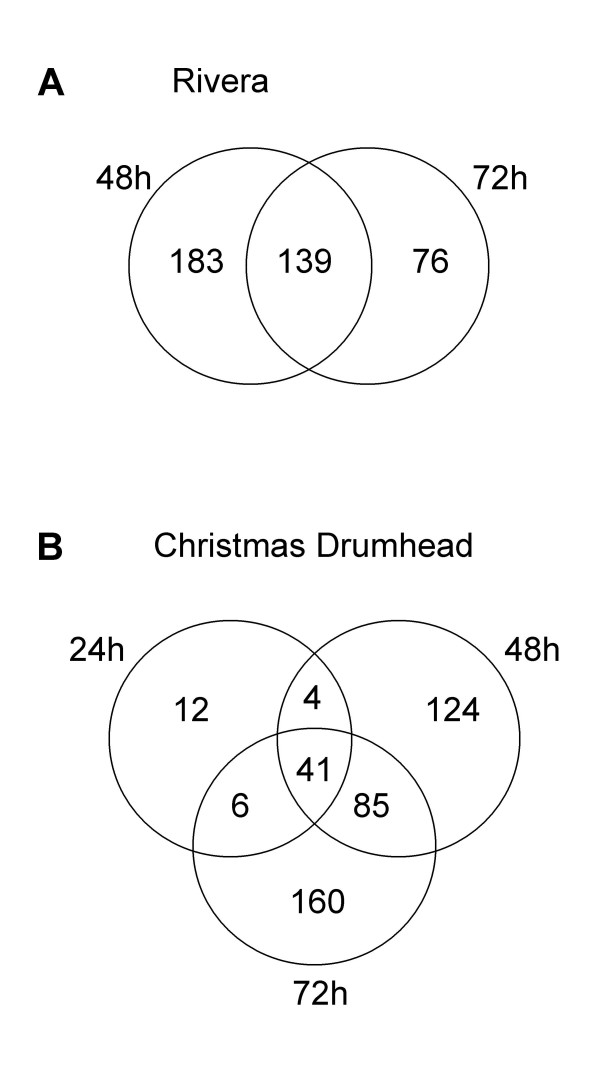
**Comparison of gene induction over time after *P. rapae *feeding in cultivars Rivera and Christmas Drumhead**. A, Venn diagram representing the distribution in Rivera of transcripts activated after 48 and 72 h of *P. rapae *challenge. B, Venn diagram representing the distribution in Christmas Drumhead of transcripts activated after 24, 48, and 72 h of *P. rapae *challenge. The numbers in the overlapping area indicate the shared number of genes in the comparisons and include genes with an average expression ratio ≥ 2-fold and a P value < 0.05 in both experiments. Numbers outside the overlapping area represent genes specifically induced at one time point.

A comparison of the genes activated at the different time points tested in Rivera showed that 43% of the genes that were induced after 48 h were still induced after 72 h of feeding (Figure 3A). In Christmas Drumhead, 65% of the genes that were induced after 24 h were still up after 48 and even after 72 h of larvae feeding (Figure 3B). This illustrates a relatively long lasting induction for a large proportion of the genes.

The observation that Rivera has a stronger direct defense but a slower transcriptional response after *P. rapae *attack suggests that this cultivar may have a higher level of constitutive direct defense. To study this, we compared gene expression levels in control plants of both cultivars. After hybridizing Rivera against Christmas Drumhead control material, using the same selection criteria as described above, we identified 15 genes with a significantly higher constitutive expression in Rivera (Table 1). However, none of these genes is clearly associated with a higher constitutive level of direct defense.

**Table 1:** Genes with a higher constitutive expression in Rivera compared to Christmas Drumhead.

**Probe identification and Putative Function**	**AGI Code**	**Number of Times Higher in Rivera**	**P value**
Expressed protein	At1g15230	9.75 ± 1.42	0.010
Kelch repeat-containing F-box family protein	At1g60570	7.67 ± 1.17	0.009
Expansin (EXP1)	At1g69530	2.49 ± 1.26	0.020
La domain-containing protein	At1g79880	4.59 ± 1.31	0.011
60S ribosomal protein L23 (RPL23B)	At2g33370	6.12 ± 1.31	0.007
Expressed protein	At2g34690	2.19 ± 1.19	0.017
Protodermal factor 1 (PDF1)	At2g42840	3.10 ± 1.58	0.050
Acyl- [acyl-carrier-protein] desaturase	At2g43710	2.06 ± 1.10	0.006
Glycosyl hydrolase family 1	At3g18080	2.40 ± 1.39	0.044
Zinc finger (C3HC4-type RING finger) family protein	At4g01023	9.75 ± 1.31	0.005
Expressed protein	At4g01220	6.48 ± 1.47	0.014
Expressed protein	At4g37440	2.41 ± 1.39	0.044
Expressed protein	At5g09980	3.90 ± 1.04	0.013
Germin-like protein (GER3)	At5g20630	2.39 ± 1.34	0.035
Expressed protein	At5g20935	5.54 ± 1.08	0.001

Relative difference in constitutive gene expression in Rivera compared to Christmas Drumhead measured in control plants. Mean expression ratios (± SE) were calculated from three biologically independent experiments. The P values denote the significant difference of the mean log-transformed ratios of unchallenged Rivera over unchallenged Christmas Drumhead plants.

### Validation of microarray data

**Figure 4:**
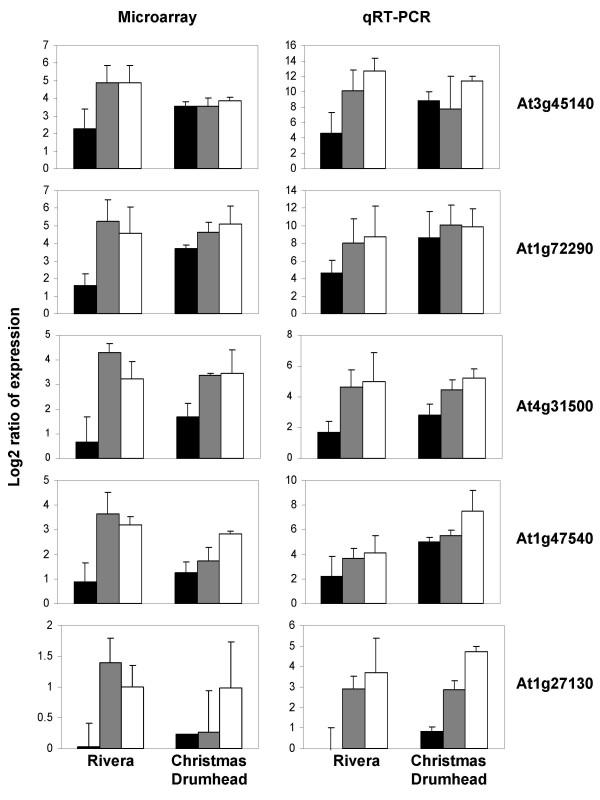
**Comparison of microarray and qRT-PCR analysis of five genes**. Log_2 _ratios of five selected genes (At3g45140, At1g72290, At4g31500, At1g47540, and At1g27130) after infestation of Rivera and Christmas Drumhead by *P. rapae*. On the left, the log_2 _ratio patterns from the microarray analysis. On the right, the log_2 _ratio patterns from the qRT-PCR analysis. Black, gray and white bars represent log_2 _ratios after 24, 48, and 72 h of *P. rapae *feeding, respectively. All bars contain their corresponding standard deviation.

To validate the microarray data, we selected five genes related to defense responses that showed high expression changes in both cultivars at one or more of the tested time points, to be analyzed with quantitative real-time PCR (qRT-PCR). Figure 4 shows log_2 _ratios of the five selected genes in Rivera and Christmas Drumhead as determined by both microarray and qRT-PCR analyses. For all genes, the log_2 _ratios were larger using qRT-PCR compared with microarray. Although fold induction in gene expression, especially for low abundant mRNAs, has been shown to differ between the two methods [26], the qRT-PCR and microarray analyses showed similar expression patterns after *P. rapae *feeding in both cultivars (Figure 4), showing the reliability of the microarray data.

### Comparison of transcriptional changes upon *P*. *rapae *feeding

**Figure 5:**
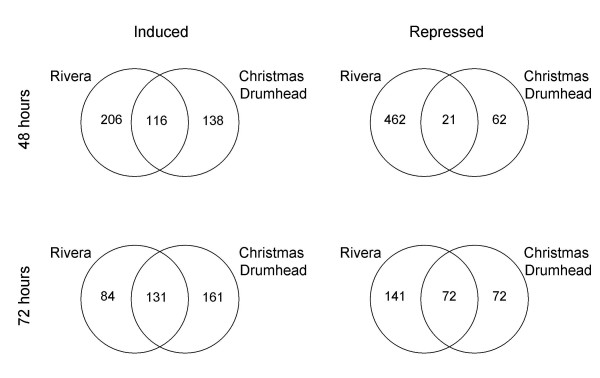
**Gene expression in cultivars Rivera and Christmas Drumhead after *P. rapae *feeding**. Venn diagrams representing the distribution of induced and repressed genes after 48 and 72 h of *P. rapae *feeding. The numbers in the overlapping areas indicate the shared number of genes in the comparisons and include genes with an average expression ratio ≥ 2-fold or ≤ 0.5-fold and a P value < 0.05 in both experiments. Numbers outside the overlapping area represent genes specifically induced or repressed in one cultivar.

**Figure 6:**
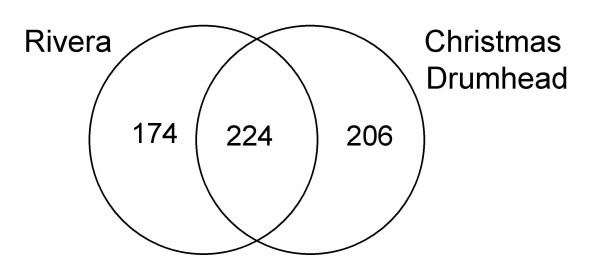
**Gene induction in cultivars Rivera and Christmas Drumhead after *P. rapae *feeding**. Venn diagram representing the distribution of induced genes when combining all time points tested. The number in the overlapping area indicate the shared number of genes in the comparisons and include genes with an average expression ratio ≥ 2-fold and a P value < 0.05 in both experiments. Numbers outside the overlapping area represent genes specifically induced in one cultivar.

To investigate which *P. rapae*-induced genes could play a role in direct defense, the overlap in transcriptional responses in Rivera and Christmas Drumhead was analyzed. After 48 h of larval feeding, 64% of the 322 induced genes in Rivera were not induced in Christmas Drumhead. Furthermore, 54% of *P. rapae-*induced genes in Christmas Drumhead were not induced in Rivera at this time point (Figure 5). After 72 h of larvae feeding, 39% of the 215 induced genes in Rivera were not induced in Christmas Drumhead and 55% of *P. rapae*-induced genes in Christmas Drumhead were not induced in Rivera (Figure 5). When comparing the overlap between transcriptional responses after combining all tested time points, the data show that 44% of the genes induced in Rivera and 47% of the genes induced in Christmas Drumhead were not induced at any tested time point in the other cultivar (Figure 6). All induced genes were classified according to their putative functional categories. Induced genes that are known to be involved in defense in *A. thaliana *are listed in Table 2. The complete list of *P. rapae*-induced genes is given in Additional file 1.

**Table 2:** Defense-related genes induced after *P. rapae *feeding in cultivars Rivera and Christmas Drumhead.

		**Rivera**			**Christmas Drumhead**		
**Probe Identification and Putative Function**	**AGI Code**	**24 h**	**48 h**	**72 h**	**24 h**	**48 h**	**72 h**

**Genes only induced in Rivera**							
Basic endochitinase	At3g12500	1.12	2.54*	2.62	1.06	1.37	1.94
Cup-shaped cotyledon1 protein (CUC1)	At3g15170	1.07	1.63	2.21*	1.11	1.26	1.92
DNA-binding protein	At1g49950	1.35	0.34	2.25*	1.66	1.81	1.50
Glutathione S-transferase	At1g27130	1.02	2.64*	2.01*	1.17	1.21	1.98
Glycosyl hydrolase 1 (BG1)	At1g52400	1.14	2.79	11.00*	2.71	-^1^	6.45
Lectin	At5g35950	0.89	2.51*	1.32	1.02	1.28	1.79
MYB transcription factor	At1g71030	1.16	2.07*	1.60	1.26	1.06	1.79
Telomere repeat-binding protein	At3g46590	0.95	2.48*	1.41	1.24	0.87	1.51
Terpene synthase	At4g16730	1.13	4.15*	2.82*	1.16	1.26	1.97
Trypsin inhibitor	At2g43520	1.19	1.74	3.70*	1.51	-^1^	2.44
**Genes only induced in Christmas Drumhead**							
Cytochrome P450 71B15 (CYP71B15)	At3g26830	1.01	1.90	-^1^	1.32	1.33	3.51*
ERF domain protein 9 (ERF9)	At5g44210	1.08	1.47	-^1^	1.19	1.23	2.01*
Glutathione S-transferase (ERD9)	At1g10370	1.04	1.55	1.33	1.54	1.77	2.10*
IAA-amino acid hydrolase 3 (IAR3)	At1g51760	1.13	1.76	1.42	1.05	1.23	2.01*
Lectin	At3g16400	1.13	1.86	1.51	1.58	2.03*	1.78
Legume lectin	At1g53070	0.98	1.73	2.03	1.31	1.67	4.21*
MADS-box protein (AGL74)	At1g48150	1.42	0.44	1.40	1.87	2.79*	1.54
Terpene synthase	At5g23960	-^1^	-^1^	-^1^	1.27	1.15	4.29*
Tryptophan synthase β subunit 2 (TSB2)	At4g27070	1.03	1.50	1.19	1.30	2.00	2.94*
Vegetative storage protein 2 (VSP2)	At5g24770	-^1^	1.10	6.96	2.68	3.49	16.20*
**Genes induced in both cultivars**							
Allene oxide synthase (AOS)	At5g42650	1.71	3.27*	2.46*	1.72	2.08*	3.50*
Coronatine-responsive tyrosine aminotransferase	At4g23600	2.28	28.34*	10.11*	7.70*	7.89*	14.70*
Cysteine proteinase (RD21A)	At1g47128	1.02	2.06*	2.83*	1.83	2.66*	3.96*
Cytochrome b5	At2g46650	1.07	3.02*	1.71	1.18	3.13*	3.68
Cytochrome P450 79B2 (CYP79B2)	At4g39950	1.47	3.23*	4.45*	1.37	1.74	7.18*
Cytochrome P450 83B1 (CYP83B1)	At4g31500	1.59	19.92*	9.38*	3.23*	10.40*	10.99*
Ethylene-responsive element-binding protein	At5g07580	1.99	6.88*	1.89	3.17*	5.73*	7.37*
Glutathione S-transferase 6 (GST6)	At2g47730	1.06	2.03*	1.50	1.44	2.82*	2.40*
Hydroperoxide lyase (HPL1)	At4g15440	1.26	2.88*	2.05*	1.51	2.86*	3.75*
Lectin	At3g16470	1.71	3.67	15.33*	3.36	5.93*	7.21*
Lectin kinase	At3g45410	2.57	14.61*	4.53*	8.38*	3.88*	7.04*
Lipoxygenase (LOX2)	At3g45140	4.74	29.91*	29.27*	11.65*	11.89*	14.53*
MYB transcription factor (MYB49)	At5g54230	1.17	4.36*	1.96	1.13	1.63	6.29*
Myrosinase-associated protein	At1g54020	1.46	4.28*	5.01*	3.06*	2.22*	6.54*
Plant defensin-fusion protein (PDF2.3)	At2g02130	1.11	1.34	2.16*	1.76	2.92*	2.19*
Polygalacturonase inhibiting protein 2 (PGIP2)	At5g06870	1.09	3.37*	5.99*	3.04	5.31*	20.16*
Terpene synthase	At1g61120	1.69	3.48*	5.32*	3.37*	2.44*	3.04*
Trypsin inhibitor	At2g43530	1.59	2.66*	4.34*	3.25	2.11*	4.62*
Trypsin/protease inhibitor	At1g72290	3.03	38.70*	23.75*	13.18*	24.37*	34.11*
Tryptophan synthase α subunit (TSA1)	At3g54640	1.19	15.18*	6.72*	2.73	17.34*	12.69*
Tryprophan synthase β subunit 1 (TSB1)	At5g54810	0.94	5.48*	3.43*	1.34	3.91*	4.47

Relative changes in gene expression after challenge with *P. rapae *larvae were measured in Rivera and Christmas Drumhead plants. Mean expression ratios are calculated from three biologically independent replicates. Only genes known to be involved in defense in *A. thaliana *are shown. *Fold change ≥ 2 with a P-value < 0.05. ^1^70-mer oligonucleotide did not hybridize in any of the three replicates. AGI, Arabidopsis Genome Initiative.

To check whether the overlap between the two cultivars was influenced by the stringency of our selection criteria, we performed statistical analyses using a 1.5-fold cut-off value while keeping the P value threshold at 0.05. With the less stringent method, 67% and 25% of *P. rapae-*induced genes in Rivera were only induced in this cultivar after 48 and 72 h, respectively. Based on these less stringent criteria for Christmas Drumhead, 55% and 73% of *P. rapae-*induced genes were induced only in this cultivar after 48 and 72 h, respectively. This indicates that the small overlap in transcriptional responses of the two cultivars is independent of threshold stringency for classifying genes as being induced.

The small overlap between regulated genes in Rivera and Christmas Drumhead does not apply only to induced genes but even more so to repressed genes. After 48 h of larval feeding, 96% of the genes repressed in Rivera were not repressed in Christmas Drumhead and 75% of the repressed genes in Christmas Drumhead were not repressed in Rivera (Figure 5). When larvae had fed for 72 h, 67% of the genes repressed in Rivera were not repressed in Christmas Drumhead and 50% of the repressed genes in Christmas Drumhead were not repressed in Rivera (Figure 5). A large proportion of the repressed genes in both cultivars are involved in photosynthesis and protein metabolism (Additional file 1).

### Role of JA in response to *P. rapae*

Several studies in *A. thaliana *have shown that a large percentage of *P. rapae-*inducible genes are under the control of the jasmonate pathway [14,16]. To get more insight into the function of *P. rapae-*induced genes and their role in defense in *B. oleracea*, transcriptional responses to *P. rapae *were compared with those triggered by the application of JA. Within the same experiment as that for *P. rapae *induction, seven-week old plants were treated with JA and leaf material was collected after 6 hours. Using the selection criteria described above, we identified 46 genes in Rivera and 80 genes in Christmas Drumhead to be JA-inducible. The complete list of JA-induced genes is given in Additional file 2. Comparison of JA-responsive genes with the *P. rapae*-induced genes revealed that less than 30% of the *P. rapae*-induced genes were responsive to JA in both cultivars. Our results suggest that *P. rapae *induced more jasmonate-independent than jasmonate-dependent genes.

## Discussion

### *Arabidopsis thaliana *oligonucleotide microarrays are applicable to *Brassica *studies

In this study, we aimed at getting insight into the transcriptional responses of two *B. oleracea *cultivars after attack by larvae of the small cabbage white butterfly *P. rapae *by using full genome microarray analyses. *Brassica *is not yet fully sequenced and microarrays based on the *Brassica *genome are not yet available. Because of this, we decided to use microarrays based on 70-mer synthetic oligonucleotides as these had been shown to minimize cross-hybridization and to be capable of recognizing related DNA sequences of *B. oleracea *[24]. Overall, 90% of the oligonucleotides present on the microarray showed intensity signals after hybridization. Additionally, for five genes the data obtained from microarray analysis were validated using quantitative real-time PCR and showed to be reliable (Figure 4). In accordance with our results and the studies mentioned above, we expect that all species within the Brassicaceae can be analyzed with *A. thaliana *based oligonucleotide microarrays. Of course, genes specific for *Brassica *will not be detected using these microarrays.

### Transcriptional responses differ between *Arabidopsis thaliana *and *Brassica oleracea*

Given that *A. thaliana *and *B. oleracea *belong to the same plant family and show high sequence identity, we expected to identify a large number of *P. rapae*-induced genes from *A. thaliana *in *B. oleracea*. Reymond and co-workers (2004) performed a study in *A. thaliana *ecotype Col-0 in which they identified 111 *P. rapae*-induced genes (≥ 2-fold induction and P value < 0.05) using a microarray representing around 7200 *A. thaliana *genes. Another study, using the same *A. thaliana *ecotype, identified 128 induced genes with at least a 2-fold induction after both 12 and 24 h of *P. rapae *feeding using a full-genome Affymetrix ATH1 chip [16]. Both studies also investigated the transcriptional response upon application of methyl jasmonate (MeJA), a volatile derivative of JA. Interestingly, when comparing the two *A. thaliana *studies, only 9% of the *P. rapae*-induced and 3% of the MeJA-induced genes identified by Reymond and co-workers (2004) were also found to be induced in the study of de Vos and co-workers (2005). The fact that both studies used the same ecotype of *A. thaliana *suggests that the induction of genes is highly dependent on the environmental and experimental conditions used. Factors that might explain the small overlap between the two studies include: (1) different time points after infestation: 3 to 5 h in the study by Reymond and co-workers (2004) versus 12 and 24 h in the study by de Vos and co-workers (2005), and (2) different larval stages: fourth to fifth larval instar in the study by Reymond and co-workers (2004) versus first to second larval instar in the study by de Vos and co-workers (2005).

In comparison with our results, 16% of the *P. rapae-*induced genes identified by Reymond and co-workers (2004) in *A. thaliana *were also induced in *B. oleracea *when combining data for significantly induced genes in Rivera and Christmas Drumhead. Thirteen percent of the genes identified as induced by *P. rapae *in the study by De Vos and co-workers (2005) were also significantly induced in our study. When focusing on the overlap between JA-induced genes in *B. oleracea *and *A. thaliana*, we found that 19% of the JA-induced genes identified by Reymond and co-workers (2004) were also induced in *B. oleracea*. Of the JA-responsive genes in *A. thaliana *identified by de Vos and co-workers (2005), 9%were also induced by JA in *B. oleracea*. In contrast to the application of JA in our study, both *A. thaliana *studies sprayed MeJA to trigger the jasmonate pathway. The use of different derivatives of JA and the difference in application might contribute to the small overlap in induced genes between the studies.

### Differences between cultivars Rivera and Christmas Drumhead

We observed differences in performance of *P. rapae *larvae that had fed for 6 days on Rivera and Christmas Drumhead (Figure 1), indicating a higher level of direct defense in Rivera. However, it is not known if this higher level of direct defense is due to constitutive or inducible mechanisms, or a combination of the two. Induced defenses in crucifers against herbivorous insects, including *A. thaliana *and *Brassica*, are well documented [27-30], indicating the presence of inducible components. We performed microarray analyses after challenging Rivera and Christmas Drumhead plants with *P. rapae *larvae and found many differences in the transcriptional response of the two cultivars. For a careful comparison of transcriptional responses, the best approach is to carry out all treatments at the same time under identical conditions. In our experiments, all conditions were kept as constant as possible: biological replicates were performed at the same time, in the same greenhouse, larvae of the same developmental stage from the same rearing batch were used, and the data were analyzed using the same statistical methods. In this way, reliable comparisons can be made between cultivars and treatments.

#### Timing

Investigation of the transcriptional responses to *P. rapae *feeding showed that both cultivars responded to the herbivore, but the responses differed in timing. The fastest activation of gene expression was found in Christmas Drumhead in which 63, 254, and 292 genes were significantly induced after 24, 48, and 72 h of caterpillar feeding, respectively (Figure 2B). Rivera, on the other hand, showed a slower transcriptional response as no genes were significantly induced after 24 h. After 48 h of larval feeding we identified 322 induced genes followed by 215 after 72 h (Figure 2A). The slower transcriptional response of Rivera did not hold for the response to JA application. Although JA induced around half the number of genes in Rivera than in Christmas Drumhead at 6 h after treatment, there is a clear induction of gene expression in Rivera. The fact that both cultivars responded to JA application at the same time suggests that the difference in timing is specific for the response to *P. rapae *larvae. However, it can not be excluded thatanydifference in timing that might existisobscured by the effect of the high concentration JA used in the experiment. Working with *B. oleracea *linesgenetically deficient in JA signaling might be more informative. At present such lines are not available. The observation that larvae grew slower on Rivera and induced a slower transcriptional response, suggests that Rivera has a higher level of constitutive defense. However, when we compared constitutive gene expression between the two cultivars, none of the genes with a higher expression in Rivera is clearly associated with a higher constitutive defense (Table 1).

#### Overall differences in transcriptional response

The transcriptional response of Rivera differed from that of Christmas Drumhead. The comparison of *P. rapae*-induced transcriptional changes among the two cultivars at 48 h revealed that 64% of the genes induced in Rivera were not induced in Christmas Drumhead and 54% of the genes induced in Christmas Drumhead were not induced in Rivera (Figure 5). After 72 h of caterpillar feeding, 39% of the genes induced in Rivera were not induced in Christmas Drumhead and 55% of the genes induced in Christmas Drumhead were not induced in Rivera (Figure 5). Because the large number of genes only induced in one of the cultivars might be an effect of timing, we also looked at the overlap between transcriptional responses by taking into account all time points. Among the genes induced at one or more of the time points in Rivera, 44% was not induced in Christmas Drumhead at any time point tested. Similarly, 47% of the genes induced after one or more time points in Christmas Drumhead were not induced in Rivera at any time point tested (Figure 6). This shows that the effect of timing does not explain the difference in transcriptional responses. Thus, the two cultivars dramatically differ in transcriptional responses to caterpillar feeding.

#### Induction of specific defense related genes

Several defense related genes are induced in *B. oleracea *after *P. rapae *feeding (Table 2). Some of these genes were specifically induced in Rivera and might therefore be involved in the stronger direct defense of this cultivar. One of these genes encodes a putative glutathione S-transferase (GST, At1g27130). GSTs are a group of stress response proteins that contribute to cellular survival after oxidative damage [31]. Another gene specifically induced in Rivera encodes a putative trypsin inhibitor (At2g43520). Trypsin inhibitors are proteinase inhibitors which provide protection against the proteolytic enzymes of herbivores [32,33].

Among the genes that were induced in both cultivars, we found some genes of the lectin family to have a higher level of induction in Rivera than in Christmas Drumhead after *P. rapae *feeding. Lectins are carbohydrate-binding proteins, many of which play a role in plant defense by binding glycoconjugates in the intestinal tract of insects [34]. Among the six *lectin *genes that were induced in both cultivars, three (At1g52070, At3g21380, and At5g35950) showed a significantly higher induction in Rivera than in Christmas Drumhead after 48 h of caterpillar feeding (Between subjects Student *t *test, P < 0.05).

Interestingly, a *terpene synthase *(At5g23960) that was induced in Christmas Drumhead after 72 h of *P. rapae *feeding was not hybridized in Rivera at any time point tested (Table 2). Terpene synthases are involved in important regulatory steps in formation of terpenes, which are volatile compounds that could attract natural enemies of the herbivore [35-39]. The *A. thaliana *homologue of the terpene synthase induced in Christmas Drumhead has been found to be responsible for the mixture of sesquiterpenes emitted from *A. thaliana *flowers [40]. Floral volatiles appear to attract species-specific pollinators, while volatiles emitted from vegetative parts of the plant, especially those released after herbivory, serve as attractants for the enemies of herbivores [41]. The induction of At5g23960 in the leaves of Christmas Drumhead and the absence of induction in Rivera suggests that Christmas Drumhead may possess a stronger indirect defense.

The expression of *Lipoxygenase2 *(*LOX2*, At3g45140) and *Allene Oxide Synthase *(*AOS*, At5g42650), which are involved in the synthesis of JA, was increased in both cultivars. The *LOX2 *gene is involved in induced indirect defense of *A. thaliana *and mediates the attraction of the parasitic wasp *Cotesia rubecula *that attacks *P. rapae *caterpillars [21].

Several genes potentially involved in glucosinolate metabolism were also found to be induced. Genes involved in the biosynthesis of tryptophan (Trp) were induced in both cultivars. *Trp synthase α subunit *(At3g54640) was induced upon *P. rapae *attack in both cultivars but the induction occurred earlier in Rivera than in Christmas Drumhead. *Trp synthase β subunit 1 *(At5g54810) was significantly induced in both cultivars, but with a longer lasting induction in Rivera. *Trp synthase β subunit 2 *(At4g27070) was mainly induced in Christmas Drumhead. Genes responsible for the subsequent oxidation of Trp to form indole-3-acetaldoxime (*Cytochrome P450 79B2*, At4g39950; *Cytochrome P450 83B1*, At4g31500) were induced in both cultivars. These glucosinolate-related genes were also induced in *A. thaliana *upon *P. rapae *feeding [14]. One gene encoding a putative myrosinase-associated protein (At1g54020) was also induced in both cultivars (Table 2).

## Conclusion

Taken together, we have demonstrated that global transcriptional responses in two cultivars of the same plant species in response to insect feeding can differ dramatically. Several of these differences involve genes that are known to have an impact on *P. rapae *performance.

## Methods

### Plant growth and treatments

Seeds of white cabbage (*Brassica oleracea *var. *capitata*) cultivars Rivera and Christmas Drumhead were germinated in potting compost (Lentse Potgrond^®^). Seeds of Rivera (an F1 hybrid cultivar) were obtained from Bejo Zaden B.V. (Warmenhuizen, the Netherlands), whereas seeds from the open-pollinated cultivar Christmas Drumhead were obtained from the Centre of Genetic Resources, the Netherlands (CGN). Plants were grown in September. Two-week old seedlings were transferred to 1.45 L pots containing the same potting compost. Plants were cultivated in a greenhouse compartment with a 16 h day and 8 h night period (22 ± 2°C). The relative humidity was maintained at 60 to 70 %. Plants were watered every other day. No chemical control for pests and diseases was performed.

Larvae of the small cabbage white butterfly *Pieris rapae *were reared on Brussels sprouts plants (*Brassica oleracea *var.*gemmifera *cv. Cyrus) in a growth chamber with a 16 h day and 8 h night cycle (21 ± 2°C, 50–70% relative humidity). Seven-week old plants of Rivera and Christmas Drumhead were infested with *P. rapae *by transferring 10 first-instar larvae to the youngest, fully expanded leaf of each plant using a fine paintbrush. At 6, 24, 48 and 72 h since the start of caterpillar feeding, a disc (diameter 2.3 cm) of the infested leaf from each of 12 individual plants was collected. Leaf discs were pooled and immediately frozen in liquid nitrogen.

An induction treatment with jasmonic acid (JA) was performed by gently rubbing the youngest, fully expanded leaf with 0.5 ml of a solution containing 5 mM JA (Sigma) and 0.1% Triton X-100 (Acros Organics) with a latex-gloved finger. The Triton X-100 was added to facilitate application to the leaf surface and absorption by the cuticle [42,43]. Despite the low pH (3.3) of the solution, we did not observe any direct effects on the leaves on which the hormone was applied. Furthermore, we treated a control group of 12 plants with 0.5 ml of 0.1% Triton X-100 (pH 3.3) alone. Material from JA-treated and control plants was collected at 6 h after treatment as described above.

The whole experiment was performed in threefold to obtain 3 biological replicates.

### Insect feeding trials

The effect of plant cultivar on *P. rapae *performance was studied using first-instar larvae. Rivera and Christmas Drumhead plants were grown as described above. Ten larvae were placed on individual eight-week old plants. Plants were placed on tablets in a greenhouse compartment (16/8 h day/night period at 22 ± 2°C) and isolated from each other by a layer of water on the tablet to prevent larvae from moving to neighboring plants. After 6 days of feeding, larvae were recollected and weighed separately to the nearest 0.01 mg. After weighing, larvae were placed back on the plants they originated from. They were subsequently monitored for development and time to reach pupation. Once a larva pupated, the date of pupation was recorded, and the pupa was collected and weighed. The whole experiment was performed in tenfold to obtain 10 biological replicates.

### Microarray hybridizations

Total RNA was isolated from material of biological replicates separately by using TRIzol reagent (Invitrogen) and purified using the RNaesy MinElute kit (Qiagen). Glass microarray slides carrying 70-mer oligonucleotide probes [44] were used in hybridizations. For target labeling, 4 μg of total RNA were linearly amplified in the presence of 5-(3-aminoallyl)-UTP using the MessageAmp™ aRNA kit (Ambion). Cy3 and Cy5 mono-reactive dyes (Amersham) were coupled to the amplified RNA (aRNA) in freshly made 0.2 M sodium carbonate buffer (pH 9.0) for 1 h at room temperature. Labeling of aRNA was monitored by measuring the Cy3 and Cy5 fluorescence emissions using a nanodrop ND-1000 UV-Vis Spectrophotometer (BioRad). Immobilization of the oligonucleotide array elements was performed as described at the manufacturer's website [44]. After applying 80 μl of hybridization mixture containing (heat-denatured) labeled targets (100 pmol Cy3-labeled aRNA from control plants and 50 pmol Cy5-labeled aRNA from treated plants), slides were hybridized for 12 h at 50°C and then washed at room temperature down to 0.05× SSC. As a control for the JA treatment, aRNA from JA treated plants (coupled to Cy3) was hybridized to aRNA from Triton X-100 treated plants (coupled to Cy5).

### Microarray data analysis

Slides were scanned separately for the two fluorescent dyes using a ScanArray™ Express HT Scanner (PerkinElmer). Median fluorescence intensities for each fluor and each gene were determined using the ScanArray Express program (PerkinElmer). Array images were checked manually to exclude spots with an aberrant shape or spots located in a smear of fluorescence from the data. Median background fluorescence around each spot was calculated and subtracted from each spot. Spots with adjusted intensities lower than half the background were manually raised to half the background to avoid extreme expression ratios. Spots where the difference between spot and background median intensity was below half the background intensity for both dyes were removed from the analysis. The resulting text files were converted by ExpressConverter ver 1.5 to generate co-coordinated MEV and ANN files. MEV files were processed through TIGR-MIDAS ver 2.18. To avoid spatial bias, Lowess (Locfit) normalization was carried out within each slide in such a way that the distribution of log ratios within each subgrid had a median of zero [45]. Normalized signal intensities were used to calculate expression ratios.

Statistical analyses were carried out using TIGR-MEV ver 3.0.3. A one class Student *t*-test on log_2_-transformed expression ratios was conducted for each experimental condition. For all of the experiments, genes with a log_2_-transformed expression ratio ≥ 1 or ≤ -1 and a P-value < 0.05 were considered significantly induced or repressed. We used the names of *Arabidopsis thaliana *homologs to identify *Brassica oleracea *genes.

### Quantitative RT-PCR

Quantitative RT-PCR analyses were performed using the same pooled samples used for microarray hybridizations. One μg of total RNA was treated with DNaseI (Invitrogen) according to the manufacturer's instructions. DNA-free total RNA was converted into cDNA using the iScript cDNA synthese kit (Bio-Rad, Veenendaal, the Netherlands) according to the manufacturer's instructions. Efficiency of cDNA synthesis was assessed by qRT-PCR using primers of the constitutively expressed gene *GAPDH *(*GAPDH*-LEFT; 5'-AGA GCC GCT TCC TTC AAC ATC ATT-3'; *GAPDH*-RIGHT; 5'-TGG GCA CAC GGA AGG ACA TAC C-3'). Gene-specific primers were designed for five *B. oleracea *genes. The corresponding AGI codes of the *A. thaliana *homologs and primers are At1g27130, LEFT 5'-ATT GGA TCA GTC CAG GTG TTG-3', RIGHT 5'-AGC TGG AAA GCT GAT GGA GA-3', At1g47540, LEFT 5'-CTG AAA GAA TAC GGA GGC AAC-3', RIGHT 5'-AAT ACC GCC ACT TAG AAT CTG G-3'; At1g72290, LEFT 5'-TGG TGA CAA GTA GCT GTG GTG-3', RIGHT 5'-TCC AAG TTA TGG GCA GTG G-3'; At3g45140 (*LOX*), LEFT 5'-CTT TGC TCA CAT ACG GTA GAA GC-3', RIGHT 5'-CCT TTG CAT TGG GCT AGT TC-3' (marker gene for JA pathway); At4g31500, LEFT 5'-CCG GAA TAT CAT AGC CAC CTA TC-3', RIGHT 5'-CCT GAA GCA ATG AAG AAA GCT C-3'. Quantitative RT-PCR analysis was done in optical 96-well plates with a MyiQ Single-Color Real-Time PCR Detection System (Bio-Rad, Veenendaal, the Netherlands), using SYBR Green to monitor dsDNA synthesis. Each reaction contained 10 μl 2× IQ SYBR Green Supermix reagent (Bio-Rad, Veenendaal, the Netherlands), 10 ng cDNA, and 300 nM of each gene-specific primer in a final volume of 20 μl. All qRT-PCR reactions were performed in duplicate. The following PCR program was used for all PCR reactions: 95°C for 3 min; 40 cycles of 95°C for 30 sec and 60°C for 45 sec. C_T _(threshold cycle) values were calculated using Optical System Software, version 2.0 for MyIQ (Bio-Rad, Veenendaal, the Netherlands). Subsequently, C_T _values were normalized for differences in cDNA synthesis by subtracting the C_T _value of *GAPDH *from the C_T _value of the gene of interest. Normalized gene expression was than obtained from the equation 2^-ΔCT^. Normalized gene expression values were used to calculate log_2_-transformed expression ratios for each experimental condition. A one class Student *t*-test on log_2 _transformed ratios was conducted for each experimental condition using TIGR-MEV version 3.0.3.

Quantitative RT-PCR products were resolved on agarose gel and genes identities were confirmed by sequencing.

## Authors' contributions

CB performed microarray experiments, data analysis and drafted the manuscript. EHP performed the insect feeding trails and the accompanying data analysis. GS assisted in the plant treatments. RV, MD and BV formulated the project and were responsible for the coordination and supervision of research and they all contributed to the writing of the manuscript. All authors read and approved the final manuscript.

## Supplementary Material

Additional file 1 Mean expression ratios of genes induced and repressed after challenge with Pieris rapae for 6, 24, 48 and 72 h in Brassica oleracea cultivars Rivera and Christmas Drumhead. Click here for file

Additional file 2 Mean expression ratios of genes induced after Jasmonic acid treatment. Click here for file
